# Innovation sustainability in challenging health-care contexts: embedding clinically led change in routine practice

**DOI:** 10.1177/0951484812474246

**Published:** 2012-11

**Authors:** Graham P Martin, Simon Weaver, Graeme Currie, Rachael Finn, Ruth McDonald

**Affiliations:** * Department of Health Sciences, University of Leicester, Leicester, UK; † Brunel University, School of Social Sciences, Uxbridge, UK; ‡ Warwick Business School, University of Warwick, Coventry, UK; § School of Management, University of Sheffield, Sheffield, UK; ** Business School, University of Nottingham, Nottingham, UK

## Abstract

The need for organizational innovation as a means of improving health-care quality and containing costs is widely recognized, but while a growing body of research has improved knowledge of implementation, very little has considered the challenges involved in sustaining change – especially organizational change led ‘bottom-up’ by frontline clinicians. This study addresses this lacuna, taking a longitudinal, qualitative case-study approach to understanding the paths to sustainability of four organizational innovations. It highlights the importance of the interaction between organizational context, nature of the innovation and strategies deployed in achieving sustainability. It discusses how positional influence of service leads, complexity of innovation, networks of support, embedding in existing systems, and proactive responses to changing circumstances can interact to sustain change. In the absence of cast-iron evidence of effectiveness, wider notions of value may be successfully invoked to sustain innovation. Sustainability requires continuing effort through time, rather than representing a final state to be achieved. Our study offers new insights into the process of sustainability of organizational change, and elucidates the complement of strategies needed to make bottom-up change last in challenging contexts replete with competing priorities.

## Background

A combination of demographic transitions, changing needs and budgetary pressures means that health-care systems worldwide are invoking major changes in the organization and delivery of services. Improving current practice is seen as an important way of addressing these difficulties, but achieving such change has proven challenging in health-care systems characterized by policy ambiguities, organizational complexities and powerful professional groups who may not favour change. A growing body of research has sought to comprehend the challenges of organizational change in health care, and assist those charged with implementing innovative ways of working.^1^ However, to date, such study has largely addressed only the initial phase of implementing change, focusing almost exclusively on the trialling and roll-out of novel approaches to care. Far less attention has been devoted to the question of how to sustain organizational change introduced with initial success: one recent review finds that ‘in most studies of change, the focus has been with the “front end”, with initiation, resistance, and implementation’;^2^ another laments the ‘near absence of studies focusing primarily on the sustainability of complex service innovations.’^3^

Recognition is increasing, however, of the fact that apparently successful initial implementation of organizational innovations – such as reconfigured roles, revised protocols and new care pathways – does not always result in sustained, longer-term change. The UK's National Health Service (NHS) Institute notes the phenomenon of the ‘improvement-evaporation effect’, whereby the benefits reaped from new practices diminish over time.^4^ This may be a particular challenge for smaller-scale, clinically led innovations, which do not enjoy the policy backing or organizational sponsorship of larger-scale changes.^5^ Whereas large-scale shifts in care can be enforced ‘top-down’ through diktat, surveillance and incentivization, ‘bottom-up’ innovation lacks such tools. Yet clinically led changes are seen by policy as crucial to ensuring health-care systems are reformed in ways that secure improved effectiveness, efficiency and responsiveness to patient need.^6,7^

The question of how to ensure that clinically led change sustains through time, rather than ‘evaporating’, is thus a crucial one, but to date has been subject to limited scrutiny. The expanding literature on the ‘front end’ of implementation, and the limited literature that goes beyond this, highlights the importance of a number of variables, including characteristics of the innovation itself,^8^ the role of team dynamics, professional involvement and alignment with existing practices,^9–11^ and – in particular – the crucial impact of the wider organizational and policy context.^2,11,12^ However, the ‘factors that need to be in place to increase the probability of changes being sustained over the medium to long term may be different from those required to prompt initial development’.^2^ Through time, favourability of initial conditions is likely to fade in importance; given the turbulence and changing power dynamics that characterize many health-care systems, other factors are likely to affect sustainability. Changing policy, and organizational and professional politics, are likely to be influential,^13^ and adaptation may thus be needed, especially in terms of the ‘soft periphery’ around innovations that ensure their fit with wider organizational environments.^14^ For Davies *et al.*,^15^ enthusiasm, reflexive practice, multiple levels of leadership, generation and use of evidence and performance monitoring are crucial to sustaining the implementation of evidence-based practice in such changing environments. However, a rather different set of challenges and opportunities is likely to face changes to the organization of care from those faced by more micro-level process change, on which the majority of the limited work on sustainability to date has focused.^8,15,16^

A gap in knowledge exists; therefore, around the nature of the challenges faced in trying to sustain and embed clinically led organizational innovations beyond initial implementation, the way in which these vary by context, and the strategies that might help overcome them. The literature is also relatively silent on the unintended consequences of sustainability: which priorities are favoured as some services are sustained but not others; whose interests do these serve; what are the opportunity costs? This paper seeks to address these gaps, drawing on comparative case-study research on service innovations in the area of clinical genetics which sought, following initial funding and implementation, to secure longer-term sustainability. (There is considerable debate in the literature as to what constitutes a ‘sustained’ service: some argue that it must be measured in terms of fidelity to the intervention as originally designed, while others draw attention to the process by which services will evolve through time, giving rise to inevitable ‘deviation’ from the prototypical intervention. There are also debates about how long a service must remain to be considered sustained [for a useful overview of these definitional debates, see Wiltsey Stirman *et al.*^8^]. Given these definitional disputes, and on account of the aim of this study to expose and elaborate issues in sustainability that might be further explored in future research, we defined sustainability heuristically and inclusively as ‘continuation of a service beyond its initial pilot funding’, making no judgements about fidelity to original intent [which, as will be noted below, was variable across cases]). Our work follows four cases longitudinally from their initial selection for fixed-term pilot funding via a national initiative, through their efforts to find follow-up funding from local sources, to their ongoing work to maintain funding and embed themselves as essential parts of their health economies.

## Setting and methods

This paper draws on two sequential studies, the first an evaluation of the national pilot initiative through which the cases were originally funded, the second a follow-up study examining the degree to which these services were sustained three-to-four years after pilot funding had ceased. In 2003, the government announced funding for 28 pilots to trial new approaches to providing clinical-genetics services in the English NHS, crossing previous organizational boundaries and involving professionals and sectors that had previously not been involved in genetics provision.^17,18^

The study team carried out a process evaluation of this pilot programme (2005–2007), covering a theoretically guided sample of 11 case studies.

The follow-up study (2011–2012) involved further research with a subsample of four of these cases, which had initially succeeded in securing ongoing local funding.^19^ Again our sampling strategy was theoretical,^20^ focusing on consistencies and divergences in several characteristics which the literature, and our earlier findings, suggested might be relevant in the path to sustainability: clinical specialty; degree to which the original innovation derived from an evidence-based model or was locally developed; lead's professional background; organizational context; and source of initial postpilot funding. Two of these variables were of particular note: organizational context (primary care versus hospital-based settings) and degree to which the innovation represented a locally developed approach to the reorganization of care. The former may have significant implications for how sustainability might be achieved (in terms of strategies, stakeholders and funding process),^21^ while the latter may be particularly important for credibility of the organizational innovation with different groups of stakeholders.^3^ These variables were given particular prominence in our sampling strategy, to facilitate comparison and contrast (see Table 1). Cases A and B were primary care-based while C and D were hospital-based; cases A and C were derived from a nationally developed model,^22^ while B and D were locally developed innovations. Subsequent to sampling, postpilot funding in one of the selected sites (Case B) was suspended, but the other three cases continued to receive funding and provide a clinical service.

**Table 1:** Characteristics of case study sites

	Organizational innovation-based on evidence-based model	Locally designed organizational innovation
Primary care-based organizational innovation	Case A Clinical specialty: cancer genetics Led by a nurse Commissioned by a Primary Care Trust (PCT)	Case B General primary care genetics Led by a general practitioner (GP) Commissioned by PCT initially (six months), then halted
	Informed by a national model, this case developed a standalone, primary care-based service to triage people at possible risk of inherited cancer, and to provide more general information to others about wider cancer risks. It received ongoing funding from its host PCT following the pilot, and was seeking to obtain funding from neighbouring PCTs to extend its provision to a wider population.	This case offered education and advice to GPs in the local area, from a GP with a special interest in genetics. Initially covering a relatively small area, following reorganization of PCT boundaries, the area putatively covered by the service increased substantially. Interim funding was provided to continue the service post pilot, but this ceased after six months and has not been reinstated to date.
Hospital-based organizational innovation	Case C Clinical specialty: cancer genetics Led by a consultant clinical geneticist Commissioned by a consortium of PCTs	Case D Other clinical specialty* Jointly led by genetics and mainstream consultants Funded through integration into mainstream service
	Informed by a national model, this case developed an integrated approach to providing risk assessment and counselling for people at possible risk of inherited cancer. It received ongoing funding from a consortium of PCTs following the pilot, and was looking at how to improve engagement of referring GPs and expand its coverage across a wider area	This service was the result of a collaboration between a specialist clinical geneticist and a specialist consultant in a related clinical field in the same hospital, who saw an opportunity to develop joint clinics that might save money and improve patient experience. Following the pilot, the service was sustained through internal reallocation of resources within the hospital

*To preserve anonymity, the clinical specialty of this site is not disclosed. It is a lower-profile clinical area than cancer

Our approach was qualitative. We conducted in-depth interviews with key stakeholders who were either involved directly in the management and delivery of the four cases, or indirectly in related services, resource-allocation or decision-making roles, starting with the former and snowball sampling towards the latter. These included clinical leads and other clinicians, referring clinicians, business managers, commissioners and service ‘champions’. We also undertook limited observation of meetings relating to the innovations (we found that in all but one site, these meetings now pertained to ‘routine’ clinical matters rather than questions of ongoing service sustainability, and so observed meetings in only one site), and analysis of relevant documents (service descriptions for internal audiences and external publicity material; minutes of meetings). In the original evaluation, 42 in-depth qualitative interviews were conducted at two time points. For the follow-up study, participants in the original evaluation were approached regarding the possibility of participating again, and further participants who had had a role in sustaining the projects were recruited via snowball sampling. Across the four cases, a total of 41 interviews were conducted in two phases for the follow-up study.

This resulted in a sizeable data-set comprising interviews collected at four time points over a seven-year period. All interviews were transcribed in full. They were analysed using an approach informed by the constant-comparative method, but with specific attention directed towards certain issues identified *a priori* and included in the topic guide. These were informed by the literature, by our knowledge of the case-study sites, and by two theoretical frameworks that provided ‘sensitizing concepts’ around the challenges of sustaining organizational change.^23,24^ Themes were thus developed both inductively and deductively, to cover issues derived from the literature, our prior work and existing conceptual frameworks, but also issues that emerged from close, repeated readings of the data sources; data were coded to these themes by SW, and were then analysed by GPM and SW, first on a case-by-case basis, and then across themes. The other authors then each analysed selected themes according to their own expertise, ensuring the validity of the initial coding and interpretation, and adding their own insights that further developed the analysis.

## Findings

Our analysis brought to light a range of issues in the sustainability of organizational innovation; here we cover the most salient. While the challenges to sustainability were largely common across cases, divergences in both the innovations themselves and the settings in which they were located meant that the degree to which these were surmountable, and the strategies that were most appropriate in achieving sustainability, varied. We consider, first, the common challenges, then the impact of contextual divergence, and finally the viability of strategies for sustainability in different contexts.

### Shifted priorities and unevidenced effectiveness: common challenges

Across all four cases, certain issues arose consistently. Particularly notable was a sense that since the original funding, clinical genetics had diminished significantly in importance for key decision-makers nationally and locally. The white paper that heralded the announcement of these services^17^ had represented a high-water mark. In the intervening period, however, other top-down priorities had displaced it, and more generally budgetary restraint and the need to ensure that ‘core’, centrally prioritized services remained viable had meant that more marginal clinical areas – including genetics and other fields – were subject to neglect. Thus

when we're in a position where it's not clear how we're going to continue to provide what everybody would regard as core NHS services, then slightly unusual developments are much less easy to make. (Clinical geneticist, Case B)

Once flavour of the month, the clinical field thus suffered from capricious national policy, which translated into conservative decisions on the part of local commissioners who needed to ensure that ‘mainstream’ functions and centrally driven priorities were funded. Clinically led service developments were trumped by central mandates.

Given this marginality, participants in decision-making positions often suggested that clear evidence of effectiveness, or ideally cost-effectiveness, would be a powerful alternative asset. Here too, however, all four cases were somewhat lacking. Again, this was due partly to the particularities of the clinical field – cost-effectiveness in genetics services being particularly difficult to evidence given the often long-term, preventive function they serve – but was also a function of the small-scale character of the services, whose low throughput and lack of managerial, accountancy or evaluation capacity made gold-standard evidence of effectiveness difficult to produce:

Whilst they are collecting data, it's difficult to demonstrate their value or the amount of money they're saving because commissioners are looking for cost savings. So whilst they're collecting data on number of people they're seeing, times they're seeing people and their demographics, it's difficult to give a precise amount of money they're saving. (Commissioner, Case C).

In part because of the particularities of the clinical-genetics field, then, but also because of features common to all kinds of small-scale, ‘bottom-up’ projects, the prospects for sustainability of the services appeared bleak – especially given the importance of evidence and a favourable policy context in innovation sustainability and spread.^3,8^ Despite this, to varying degrees, the cases were able to convince decision-makers that they were worth sustaining. But as we discuss next, their ability to do this was heavily influenced by their divergent characteristics and contexts.

### Making a case in a challenging environment: contextual divergence

Though lacking ‘gold-standard’ evidence of their effectiveness or cost-effectiveness, service leads and their colleagues deployed a range of other arguments for the value of their work. For example, stakeholders in Case A drew attention to the national award the service had won – and the furore that withdrawing funding from a prize-winning service might attract. Case C's leads highlighted findings from audits that appeared to suggest good performance (though without controlled, ‘research-grade’ evidence to support this claim):

We won a national award which […] helped as well, plus because we've done patient evaluations, I think the patient's voice has quite a big say in what goes on. If we can prove that patients like the service and that the service has done them good, then I think that will create advocates for the service. (Lead nurse, Case A).

There was a […] national review of the services and [Case C] was at the top or was very near one of the top ones in the country and so certainly there was an element of, ‘Are you honestly going to consider decommissioning the day after a report comes out that says this is the best in the country?’ […] That, I think, probably played into some of our thinking about, well OK, there is insufficient information at this moment in time either way, therefore, the status quo is to keep funding the service. (Business manager, Case C).

Cases B and D offered similar cases for continuation based on patient-satisfaction surveys and claims about superior quality of care. Across all four cases, these claims fell well short of constituting clear-cut clinical evidence and robust economic evaluation, but sometimes they held considerable sway. This varied, however, according to two notable contextual factors across the case-study sites.

#### Degree of external dependency

While the case for sustaining Case D's service appeared no stronger than those of the other three sites, the resultant path to ongoing funding was considerably more straightforward here. This seemed, in part, due to the rather less complex organizational context faced by this service, which was located within an existing department of a hospital and involved reorganizing the way patients with a possible inherited condition were seen. There was no change in the patient group referred to the hospital; rather this was an internal reorganization that could be presented as a rational redistribution of resources within an autonomous organizational unit. Inconclusive data thus met with surprisingly little resistance:

We've changed the way people think, […] purely based on delivering a good service that works, that the patients certainly enjoy, the outcome measures are good, diagnostic yield goes up and it's not had the effects that some people predicted of taking the key group of patients out of general medical clinics into a different clinic. So I think it's worked on the basis that people can see that the clinic has worked. (Consultant clinical geneticist, Case D).

In contrast, Cases A, B and C all faced more complex organizational contexts in which a much greater variety of interest groups had to be placated – especially Cases A and B, in the relatively open system of primary care.

#### Organizational influence of service leads

Related to this, the differing roles and positions of the service leads across the four cases impacted notably on their ability to make the case for the ‘value’, broadly defined, of their services. In Case D, the service leads – physicians with considerable decision-making clout and control over resource allocation – had a relatively easy task: ‘we were sufficiently well known, sufficiently senior and sufficiently motivated to say, “We are going to do this, and it doesn't matter what on earth you say, we're going to do it”’ (Consultant clinical geneticist, Case D). Case C's lead was also a consultant, but he enjoyed less influence among the heterogeneous organizations affected by the revised care pathway his service had introduced. Cases A and B were led by primary care-based practitioners – a nurse and a general practitioner (GP) respectively – with considerably less influence within and beyond their organizations than their hospital-based counterparts, and comparatively loose links to other stakeholders with decision-making and resource-allocation responsibilities.

To varying extents, therefore, leads in Cases A, B and C faced a somewhat more challenging task in asserting their services’ value and ensuring their continuation. In the next section, we discuss how this contextual variation, and the stakeholders’ responses to it, played out in terms of comparative sustainability, highlighting how far the approaches deployed were successful in overcoming the challenges faced in these divergent contexts.

### Achieving sustainability in varying contexts: finding appropriate strategies

Lacking prioritization or cast-iron clinical-effectiveness evidence, facing contexts of varying complexity, and with leads of varying degrees of positional power, the cases deployed a number of strategies to assure their futures. Four aspects of their work seemed particularly important in this process, summarized below and in Tables 2–5.

**Table 2:** The role of networks of support in the four cases

Finding	Illustrating data
Extensive networks of clinical and managerial ‘champions’ can aid sustainability despite challenging contexts	*Case A*: ‘My role was to scope out whether there was a need for it—which was interesting because defining a need for a genetic service wasn't the easiest thing in the world, because if you crunch empirical research or demographic data or programme budget data, you won't necessarily come to the conclusion that you need a genetic service. […] So I do tend to step in every now and again. I'm not suggesting I step in to protect it, but I think services like that that are not tariff-based services, they're not protected by patient flows and choice and all that sort of stuff and market forces. They do need a different level of support and leadership to bring it to the forefront of decision makers’. (PCT director)
	*Case C*: ‘The trust have used those experiences of peers and then got far more sophisticated. […] They made sure that we had [information on the service] ahead of us, and then made much more active use of clinicians directly. So if there was an issue, they will get the clinician in, get the clinician to talk about the pressures and the issues rather than just the usual management faces, which is effective, because it allows you to have a proper dialogue and helps you understand’. (Commissioner)
	‘If we hadn't had a key helpful person from this trust [we might have faced difficulties]. So there was a relatively junior business planner who was […] keen to do things, and I think that was key as well. So when you're negotiating through third parties, if you've got a relatively jellyfish-like third party, you haven't got a hope in hell. If you've got someone who's engaged, who's listening and helping, then that's much better. I would say that is absolutely fundamental to our sustainability’. (Service lead)
Without such networks, it is difficult to make a strong case for sustainability	*Case B*: ‘The service was popular with a small set of practices and clinicians.’ (PCT general manager)
	‘It would be a concern if we were failing our patients by not providing this particular service, but I haven't heard them shouting’. (PCT senior clinical manager)
	‘I think what [the service] really needed to succeed, and perhaps didn't have, would have been from this department a very strong sense of, “Yes, this is needed and we need to push the PCTs to continue funding this”’. (Consultant clinical geneticist)

**Table 3:** Embedding in clinical systems as a means of securing sustainability

Finding	Illustrating data
Localized changes, with minimal impact on wider clinical systems or resource-allocation decisions, may be sustained relatively easily	*Case D*: ‘The long term viability is very, very strong, it's viewed as an essential service, the patients need to be seen by a [clinical specialist in the mainstream clinical area] and they need to be seen by a geneticist, so they either come to our clinic or they get pushed back into the existing clinics. […] I think we're probably pretty cost neutral for the trust, the activity we're doing is probably no different to what would be happening in the clinic and we're probably a little bit more economically efficient I would guess’. (Mainstream consultant clinical specialist)
Where impact on wider clinical systems and resource-allocation decisions is present, embedding the organizational innovation into the wider clinical system may secure its sustainability	*Case C*: ‘We've got an established service that everyone knows about, and the route for all these patients is through [Case C service], so there isn't any gynaecologist messing around—in my view, that's what I would have called it, they'd be messing around with patients who had been referred by their GP. Now these patients are all passed through [the service] before they come to gynaecology. So they may still come [to me], but they come with expert information upfront about the risk and the options that they've got available’. (Gynaecological oncologist in referring department)
	‘In the end [our case to the commissioners] was more, ‘If you don't fund this there's going to be a great black hole that no-one can pick up’. I think that's how it was funded in the end’. (Lead nurse)
Services that are not embedded into the wider system may be seen as supplementary rather than as a core part of provision, and are more vulnerable to decommissioning	*Case B*: ‘The money wasn't there, there wasn't identified any money, but I think there's also that it wasn't priority for the Primary Care Trust, nobody had any targets to reach for genetics, there was no ‘must dos’ about genetics and so it was easy for it to slip down the radar. […] The priorities were the national priorities to do with waiting times, with, cancer, for heart disease, for prevention, so there were quite a large number of priorities for any Primary Care Trust, we didn't have unusual priorities other than the national priorities in health. And there were so many, there still are, but there were so many it's quite hard to do anything other than to meet the priorities’. (PCT former senior clinical manager)

**Table 4:** The need for flexibility and responsiveness to changing contexts

Finding	Illustrating data
In areas of service delivery marginal to policy priorities, ‘tying into’ wider, persistent policy agenda can facilitate sustainability	*Case A*: ‘Our cancer survival rates are worse than our cardiology survival rates at the moment. Our female lung cancer deaths were higher than the breast cancer deaths last year. The prevention strategy is around that, about smoking cessation, exercise and reduction in alcohol intake. Not necessarily all linked to family-history services. But they all need to work together. So it's like family-history service working with the public health team, working with the health advisers that are also part of the community provider arm’. (PCT cancer services improvement lead)
Changing priorities, personnel and organizational boundaries pose threats to sustainability	*Case A*: ‘We've tried embedding it at the cancer network so the other towns would pick it up, but each PCT has their own agenda. One out of our two neighbouring PCTs doesn't have cancer as one of their priority areas. And they've got a similar population and it's like, “How can you not have it as a priority?”’ (Service manager)
	*Case B*: ‘When [Greater Beeville PCT] took over those responsibilities, first of all there was a big reduction in commissioning management capacity and secondly, a shift in priorities. […] [Greater Beeville PCT's medical director] probably had very limited awareness of [Case B's] existence during the three years that it was in place, but I think if it'd remained in [Beeville City] PCT where the medical director had signed the application, then there would have been a more active programme of canvassing and support and awareness raising which would have potentially resulted in a different decision’.
	‘It was based very much in [Beeville City] and used by GPs, but not all [Beeville City] GPs. And so it wasn't available to GPs in [western Beeville] or [northern Beeville]’. (PCT senior clinical manager)
These threats can be dealt with through flexibility in scope and delivery of service	*Case A*: ‘The mistakes I've seen a lot of services make is that they try really, really hard to establish because they think there's a need to convince people there's a need to get funded they start seeing stakeholders, but then it stops. And they think, “Well that's good enough, once we've got off the line and we're established, that's all we need to do”. But it isn't, that's actually when it starts. And the difference, between the ones who've got longevity and value in comparison to those who get decommissioned and don't have perceived value’. (PCT director)

**Table 5:** Overcoming inertia to secure sustainability in the longer term

Finding	Illustrating data
Short-term ‘fixes’, such as agreements to provide time-limited funding or fixed levels of income, offer medium-term sustainability by reducing risk to commissioners and providers	*Case C*: ‘The commissioners haven't pushed us down a very strict route of providing information activity on a monthly basis like we would do for some of the normal acute activity. So we've done very little really. […] You could say it's a lack of interest by commissioners. It's probably also because it is funded on a block contract. If it was funded on a cost-per-family or a cost-per-patient-seen or that type of thing, obviously we would have to provide the information on a monthly basis, because we wouldn't get the money following the service. So I think probably because it's based on a block contract and probably because the service itself isn't screaming and saying actually we need more money because we've got too many referrals coming in, it's probably had less scrutiny’. (Planning and commissioning manager)
In the longer-term, however, such agreements can stifle services’ ability to develop and may leave them to stagnate: in a competitive health-care system, services must continue to innovate to remain viable	*Case A*: ‘In the grand scheme of things we don't cost an awful lot of money, and if they actually rolled our service out properly across the sector which would include [neighbouring areas], we would actually be very-cost effective. But it's whether that process will take place’. (Lead nurse)
	*Case C*: ‘We've spoken to the lady in finance they don't see it being a problem. It's the same as anyone now, there's cuts everywhere. Because we're a very small service we, you know, there's a threat that we might be cut, but once again because we're a very small service and we don't cost very much and that's in our favour as well’. (Lead nurse)

#### Networks of support

For those cases facing more complex organizational contexts, with multiple stakeholder groups from across health-care sectors, a network of ‘champions’ and ‘sponsors’ from different clinical and managerial backgrounds was crucial. Whereas Case D's leads could act relatively autonomously in securing their service's future, requiring only the acquiescence of close colleagues with whom they shared high status, those projects working across care pathways required wider support. Though ultimate decisions about ongoing funding usually rested with commissioners, support from clinical colleagues could be crucial in securing recognition of the services’ wider ‘value’. Similarly, support from champions in senior managerial positions could help to secure the necessary influence to sustain the services despite lack of evidence and central policy push (Table 2).

By contrast, an absence of such a network of clinical and managerial supporters could deal a major blow to plans to sustain provision. Case B – whose funding was halted while the follow-up study was being planned – enjoyed the endorsement of far fewer champions, and interviews with those in decision-making roles suggested this had an important impact on its prospects (Table 2). Securing influence in resource-allocation processes involved considerable ‘political’ work on the part of service leads, then, to ensure that where policy push was lacking and a clear-cut evidence-based case could not be made, alternative arguments were acknowledged.

#### Clinical embedding

Alongside networks of support, position within the substantive network of clinical care was also important for sustainability. Case D, as we saw above, involved a relatively localized change to a self-contained service, which proved relatively easy to justify. In the other cases, the organizational innovations had important implications for other services, and a key task was ensuring an appropriate interface with adjacent parts of the care pathway. Where services could be readily integrated into wider clinical systems, their sustainability seemed more assured – partly because this helped to secure stakeholder endorsement noted above, and partly because they became difficult to decommission without significant knock-on effects for the rest of the system (Table 3).

Here again, Case B suffered because – as a service that involved consultative advice and education from a GP with a special interest in genetics rather than a triaging or treatment step on a care pathway – its function was never so embedded. Consequently, it constituted an ‘adjunct’ rather than a central part of provision, and its position seemed vulnerable to changing priorities and budgetary reductions. Rather than a core service, ‘it was almost a luxury that wasn't required’ (PCT senior manager, Case B; see also Table 3).

#### Responding proactively to change

As noted above, all four cases faced contexts in which the profile of clinical-genetics services had declined substantially. Cases A and C, however, were cancer-genetics services, and this allowed them to ‘tie into’ a perennial national priority (Table 4).

Other contextual changes were harder to deal with. In an NHS characterized by continual reorganization,^25^ the two primary care-based cases in particular faced a turbulent organizational environment, in which the populations they covered and the priorities they had to address were subject to ongoing change. In Case B, organizational-boundary changes resulted in a sudden shift in the size, and health needs, of the patient group served—and also a change in management personnel, which together undermined the service's case for sustainability (Table 4). Case A faced similar shifts in the primary-care environment, but here the network of stakeholders noted above helped to cushion their impact. Here too, though, changing audiences and patient populations could prove difficult to appeal to, as the service found seeking to expand provision beyond its original boundaries (Table 4).

#### Overcoming inertia to become a ‘viable risk’

Ironically, for the three services that managed to some degree to sustain themselves, one threat to their further development was becoming a taken-for-granted part of the system, rather than a project requiring continued nurture and development. As seen above, Case A struggled to expand its service beyond the area initially covered. Its host organization suspended efforts to increase its market share, settling on its original boundaries, but in an NHS characterized by increasingly market- and performance-based governance, this inertia seemed to pose risks to the longer-term ability of the service to adapt to a changing environment (Table 5).

Similarly in Case C, a commissioning decision to fund the service through a block grant had, paradoxically, simultaneously offered security to the service, and risked stifling its further development. Block funding, rather than payment according to throughput, limited the risk to both commissioner and provider by fixing a sum to secure ongoing provision of the service. As a consequence, the service's short-term future seemed relatively secure, as commissioners subjected it to less critical scrutiny. On a longer-term basis, however, there was concern that such inattention might ultimately result in stagnation, as opportunities to expand and respond flexibly to new needs were squandered (Table 5).

## Discussion and conclusion

Our study highlights a range of important issues in the sustainability and embedding of organizational innovations in a field –clinical genetics – which shares many characteristics with other, relatively marginal areas of health-care provision,^21^ where clinically led change lacks the backing of push from the centre. We show how the relationship between the nature of the services themselves, the circumstances they face and the strategies for sustainability they deploy contributes to services’ ongoing prospects. We cannot, given the nature of our study, suggest with confidence which of these are most important, or differentiate between ‘necessary’ and ‘sufficient’ conditions for sustainability. Rather, our analysis suggests that these are contextually contingent, and surfaces the important interactions between setting, leadership, the contribution of wider stakeholders and the nature of the innovation to show how, in challenging circumstances and varying contexts, those leading innovation might work to secure sustainability.

One notable finding is that sustainability cannot be viewed as a binary state. Rather, it is better understood as a continuum, and requires continuing effort. In all the sustained services there was a sense that sustainability remained a live project; while they had made varying degrees of progress towards embedding themselves in their local health-care systems, in no case could the sustainability achieved be said to be a final, steady state. In some ways this is to be expected in an organizational context in which commissioners are increasingly expected to demand strong performance, flexibility and continual improvement of providers, and ‘cosy’ commissioner–provider relationships are to be banished to the past.^7,11,26^ However, there seemed a risk that, as small services in a system undergoing big changes, they could find themselves marginal to the concerns of key decision-makers. While potentially advantageous in the short term, in the longer term it could result in stagnation due not to complacency or inflexibility, but to the uncertain dynamics of the wider environment in which they were vying for attention.

In such a context, the study suggests that some of the factors known to be important in the initial implementation of new services are more critical than others in sustaining change. Compared with the earlier literature – which, as noted above, focuses on more micro-level change^8,15^ – our meso-level focus suggests a somewhat different range of important influences. For example, of the five critical success factors identified by Davies *et al.*,^15^ enthusiasm and effort, interprofessional collaboration and multiple forms of leadership seem important, whereas evidence generation and use and performance monitoring seem rather less so. This is undoubtedly in part a product of differences in the nature and scale of the innovations under study, though as we discuss below, it may also relate to problems and ambiguities of the nature of ‘evidence’, especially in the particular context we studied. Evidence – in the narrow sense of robust information on clinical and cost-effectiveness – does not seem essential to sustaining meso-level change.^8^ In its absence, in the challenging organizational and policy context described above, leads must undertake extensive political work to ensure that broader notions of a service's ‘value’ are acknowledged and accounted for by those in decision-making positions.^10^ This meant, in these four cases, developing a range of discourses of ‘value’ embracing notions such as patient satisfaction, quality of care and external esteem. On their own, though, these were of limited persuasive power. Each of the cases also had to ensure the development of a network of support from a range of stakeholders in clinical and managerial roles,^27,28^ active work to ensure alignment with existing organizational arrangements such as care pathways^29^ and flexibility in the face of a volatile organizational context.

This also implies that sustainability of organizational innovations cannot be understood simply in terms of continued ‘fidelity’ to newly introduced practices. At the level of organizational change, it is evident that flexibility and responsiveness to wider pressures – the capacity to continue to adapt to current and foreseeable system conditions’^30^ – is a key ingredient in sustainability through time. Sustainability thus appears to be a moving goal, in which the task of maintaining clinically led change must be made compatible with the need to respond to changing expectations and priorities from external stakeholders. On the one hand, this might be taken as evidence of a healthily adaptable, patient-focused system that is capable of responding to changing needs and expectations. On the other hand – and given the points above about the importance of networks of support in situations where cast-iron evidence of cost-effectiveness is impossible to secure – it might suggest that commissioning remains a worryingly weak process that is vulnerable to provider ‘capture’.^31^ A policy context which is increasingly focused on securing value and cost-effectiveness may necessitate greater flexibility in what such services provide – or, alternatively, a chameleon-like ability to appeal to the right audience at the right time. Ensuring that commissioners have the necessary information, skills and resources to drive change will be an important task as the commissioning system in the English NHS is once again reorganized.

Such arguments aside, our study offers some important implications for managers and clinicians involved in the sustaining of new service models (Table 6). Our study offers novel insights into the multiple dimensions of this process, especially in relation to the understudied level of meso-scale, organizational changes. Moves to promote innovation adoption as a means of improving quality and containing costs are afoot worldwide; furthermore, the increasing use of competitive health-care markets internationally means that these lessons are likely to increase in importance in the future. Ongoing shifts in boundaries and accountabilities in health care are a prominent feature of health-care reforms in many countries, and consequently finding the right champions and responding to fluid expectations seem particularly important. While our findings raise important questions about the political and social nature of the sustainability process, for the enterprising health-care manager, drawing on the range of strategies we discuss may be crucial in securing, sustaining and embedding organizational change.

**Table 6:** Implications for health-care managers

This study adds to existing knowledge about the factors influencing sustainability of health-care service change and the interactions between them, especially at the level of clinically led, meso-level organizational change, where prior research is limited
Sustaining service innovations is not a one-off task: rather, it requires continuing work to ensure that services remain responsive to changing policies, priorities, populations and managerial and clinical stakeholders
While ‘bottom-up’, clinically led services may struggle to provide robust evidence of clinical and cost-effectiveness, this is not always fatal; a range of other notions of ‘value’ may hold sway in making cases for ongoing funding
In making these cases, organizational context is a crucial variable; more complex organizational contexts (such as primary care) require a variety of nuanced strategies for sustainability
Sustainability can be facilitated through the nurturing of networks of clinical and managerial champions, through embedding of services in care pathways in a way that renders them an integral part of service provision, and through alertness and responsiveness to shifting agenda and expectations
